# Dendritic and Axonal Propagation Delays Determine Emergent Structures of Neuronal Networks with Plastic Synapses

**DOI:** 10.1038/srep39682

**Published:** 2017-01-03

**Authors:** Mojtaba Madadi Asl, Alireza Valizadeh, Peter A. Tass

**Affiliations:** 1 Institute for Advanced Studies in Basic Sciences (IASBS), Department of Physics, Zanjan, 45195-1159, Iran; 2 Institute for Research in Fundamental Sciences (IPM), School of Cognitive Sciences, Tehran, 19395-5746, Iran; 3 Institute of Neuroscience and Medicine - Neuromodulation (INM-7), Research Center Jülich, Jülich, 52425, Germany; 4 Stanford University, Department of Neurosurgery, Stanford, CA, 94305, USA; 5 University of Cologne, Department of Neuromodulation, Cologne, 50937, Germany

## Abstract

Spike-timing-dependent plasticity (STDP) modifies synaptic strengths based on the relative timing of pre- and postsynaptic spikes. The temporal order of spikes turned out to be crucial. We here take into account how propagation delays, composed of dendritic and axonal delay times, may affect the temporal order of spikes. In a minimal setting, characterized by neglecting dendritic and axonal propagation delays, STDP eliminates bidirectional connections between two coupled neurons and turns them into unidirectional connections. In this paper, however, we show that depending on the dendritic and axonal propagation delays, the temporal order of spikes at the synapses can be different from those in the cell bodies and, consequently, qualitatively different connectivity patterns emerge. In particular, we show that for a system of two coupled oscillatory neurons, bidirectional synapses can be preserved and potentiated. Intriguingly, this finding also translates to large networks of type-II phase oscillators and, hence, crucially impacts on the overall hierarchical connectivity patterns of oscillatory neuronal networks.

Spike-timing-dependent plasticity (STDP) is a mechanism that modifies synaptic strengths based on the relative timing of pre- and postsynaptic spikes^1^,^2^,^3^,^4^,^5^. Whenever the presynaptic spike precedes the postsynaptic spike, STDP causes a potentiation of the corresponding synaptic strength, and a depression in the opposite case^6^. STDP is a local mechanism since the synaptic modification depends only on the spike timing of two neurons connected by a corresponding synapse. However, global structures emerge by implementing the local STDP rule in recurrent networks of spiking neurons, as revealed in numerous studies in recent years^7^,^8^,^9^,^10^,^11^,^12^,^13^,^14^,^15^,^16^,^17^,^18^,^19^,^20^,^21^. However, these computational results are in some cases incompatible with experimental observations^7^,^19^,^22^. First, the temporal asymmetric shape of the STDP window leads to the elimination of strong recurrent connections between any two neurons and also larger polysynaptic loops^7^,^21^,^23^, at least in the absence of noise^24^. Although this interesting property can explain the emergence of feedforward networks^2^,^25^,^26^, it is in contradiction to the prevalence of recurrent connections between pairs of neurons in cortical networks^27^,^28^. Second, STDP inherently is an unstable process, since it provides a positive feedback interaction among synaptic modification between two neurons and changes in their relative spike times, i.e. the more stronger the connection from neuron 1 to neuron 2, the more likely neuron 2 will fire shortly after the firing of neuron 1, leading to more potentiation of the corresponding synapse. The same argument can be brought forward for the depression of the synapses, and taken together, STDP leads to a bimodal distribution of the synaptic strengths when hard boundaries limit the upper and lower values of synaptic strengths^2^,^29^,^30^. This result also does not comply with the unimodal distribution of cortical synaptic efficacies reported for cortical networks^28^,^31^. Several variations of the STDP rule have been proposed in recent years and each of them amend some of the inconsistencies between the spike-timing based plasticity models and experimental results^2^,^9^,^19^,^29^,^32^,^33^,^34^.

The functional effects of STDP, and in particular its relation to the synchronization in neuronal networks have also led to contradicting results. Early studies on the effect of STDP showed that it enhances network synchronization through promoting causal links^26^,^35^,^36^. However, it has been shown that considering propagation delays in the models, firing in synchrony induces long-term depression (LTD) and decouples the neurons^37^,^38^,^39^. Later, an intermediate effect has been reported showing that in the presence of propagation delays, STDP promotes the self-organization of recurrent networks into mixture states at the border between randomness and synchrony^12^. This latter result depends mainly on the imbalance of potentiation and depression for small time lags between presynaptic and postsynaptic spikes, and a small difference between dendritic and axonal propagation delay times, assuming the axonal delays are larger^40^. These studies highlight the importance of forward and backward propagation delays in the functional and structural outcome of STDP. In fact, in neuronal networks delays are crucial for the emergence of different types of dynamical regimes and mechanisms^41^,^42^,^43^,^44^, e.g. for delay-induced optimal synchronization^45^,^46^,^47^,^48^,^49^,^50^,^51^.

Pre- and postsynaptic spikes arrive at the synaptic site after dendritic and axonal propagation delays, respectively; therefore the effective time lag at the synapse would be different from that of the cell bodies (more precisely at the axon hillock) and is determined by the time lag of spikes in the cell bodies and the difference of dendritic and axonal propagation delays (see Fig. 1). On the other hand, from the theory of delayed coupled oscillators it is well-known that the total propagation delay, i.e. the sum of the dendritic and axonal propagation delays, determine the synchronization tendency of the coupled neuronal oscillators^52^,^53^,^54^,^55^,^56^, which is predicted by their phase response curve (PRC)^57^,^58^. Indeed, the propagation delay and PRC of the neurons determine whether the synaptic connection is synchronizing or desynchronizing^52^,^53^,^54^,^55^,^56^. Therefore dendritic and axonal propagation delays play a dual role in the networks of coupled neurons when the synapses are modified through STDP: Their difference enters into the synaptic modification rule and their sum determines the synchronizing/desynchronizing nature of the connection^59^. Since the evolution of synaptic strengths through STDP is a slow process in comparison to the timescale of pairwise spiking interactions of the neuronal network^60^, on the timescale of a few periods of a neuronal oscillator the synaptic efficacies can be taken as constants and the theory of delayed coupled oscillators can be applied to determine the phase lag of firing of the neurons in the stable phase-locked mode^56^,^61^. Given the PRC of the neurons, this phase lag depends on the total propagation delay and the current values of synaptic efficacies. The resulted phase lag, along with the difference of the dendritic and axonal propagation delays, determines the modification of the synapses subsequently.

Based on this argument, in this study we explore how dendritic and axonal propagation delays determine the final configuration of a pair of bidirectionally coupled neuronal oscillators. We provide a general theoretical framework by assuming that the neurons are phase-locked with a phase lag which is determined by the temporary values of the synaptic constants, propagation delays, and the PRC of the neurons, and explore how the final configuration of the system can be predicted. We show that in the presence of dendritic and axonal propagation delays, the conventional pair-based additive STDP may lead to both unidirectional and bidirectional connections, or decouple neurons by weakening the reciprocal connections in both directions. Previously, it has been shown that in the presence of noise, bidirectional connections can be potentiated when in the STDP profile for small time lags potentiation dominates depression^7^,^12^,^19^,^20^,^21^,^22^,^23^,^24^,^40^. Our results show that the bidirectional connections can be preserved and potentiated even in the absence of stochastic inputs and with a balanced profile of STDP. Furthermore, commonly it is believed that STDP leads to depression of reciprocal synapses when the two-neuron dynamics are uncorrelated, and depression dominates in the STDP profile. Here we show that simultaneous depression of both reciprocal synapses is even possible in the phase-locked state (with highly correlated firing of two neurons) with a balanced STDP profile, when propagation delays are taken into account. Finally, by numerical simulations we demonstrate how our theory developed for the two-neuron motif can even be applied to recurrent networks of spiking neurons. We show that our results have significant implications to the hierarchical organization of connectivity patterns in networks of oscillatory neurons.

## Results

### Theoretical framework

**Figure 1:**
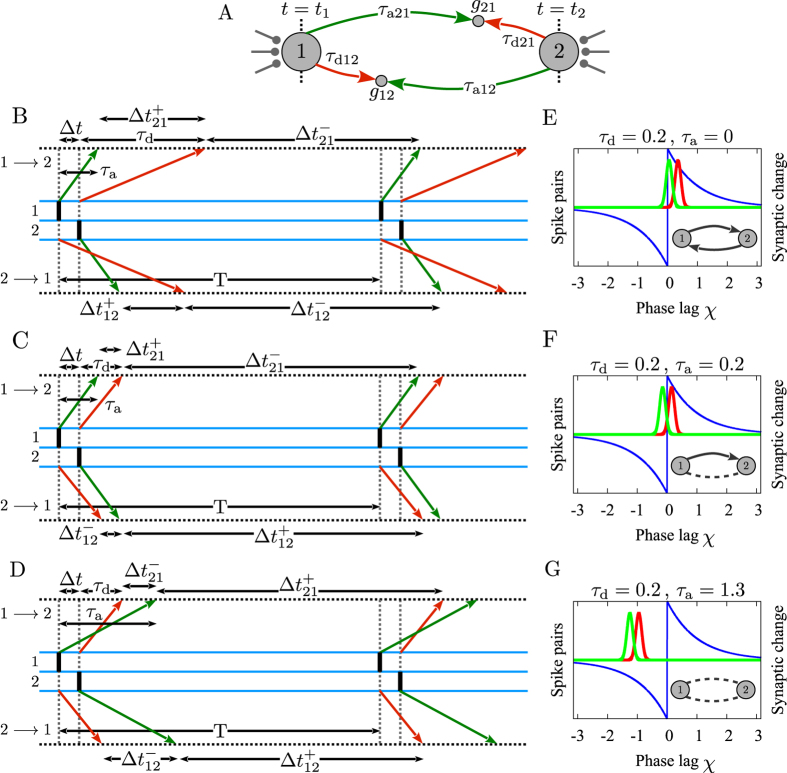
Possible synaptic modifications of two interconnected neurons fire nearly inphase in the presence of dendritic and axonal propagation delays. (**A**) Two representative identical neurons which are reciprocally coupled by initially symmetric excitatory synapses. *t*_1_ and *t*_2_ are the exact spiking times of neurons 1 and 2, respectively. *g*_*ij*_, *τ*_d*ij*_ and *τ*_a*ij*_, *i, j* = 1, 2, *j* ≠ *i* denote synaptic strength, dendritic and axonal propagation delays of the synapse *j* → *i*, respectively. (**B–D**) Assuming dendritic and axonal delays are identical in both directions i.e. *τ*_d21_ = *τ*_d12_ = *τ*_d_ and *τ*_a21_ = *τ*_a12_ = *τ*_a_, 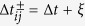 is the effective time lag for which STDP causes synaptic potentiation (upper)/depression (lower sign) of the corresponding synapse (horizontal dotted lines). T is the period of the spiking of the neurons. The vertical bars in the middle triple lines indicate spiking of the neurons. Green and red arrows represent the time of the transmission of the spike of the presynaptic neuron and the backpropagated potential of the postsynaptic neuron to the synaptic site, respectively. (**B**) Potentiation of both synapses in case *τ*_a_ < |Δ*t* − *τ*_d_|. (**C**) Formation of unidirectional connection when |Δ*t* − *τ*_d_| < *τ*_a_ < |Δ*t* + *τ*_d_|. (**D**) Depression of both synapses in case that |Δ*t* + *τ*_d_| < *τ*_a_. (**E–G**) Illustration of corresponding synaptic modifications based on a balanced STDP profile and the schematic Gaussian distribution of pre (green)- and postsynaptic (red curve) spike times for different exemplary values of dendritic and axonal propagation delays used in our simulations.

We considered two neurons described in the phase reduced model (see Methods) characterized by firing frequency *ω*_*i*_, *i* = 1, 2 and infinitesimal phase sensitivity *Z*(*φ*), coupled via delayed connections of strength *g*_*ij*_ with delay *τ*_*ij*_:


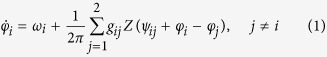


where *ω*_1_ = *ω*_2_ = *ω* and *ψ*_*ij*_ = *ω*_*i*_*τ*_*ij*_ is the rescaled delay (see Fig. 1A). The neurons fire every time their phase passes multiples of 2*π*. We assume that the propagation delay is the sum of dendritic *τ*_d_, and axonal delay *τ*_a_, that is *τ*_*ij*_ = *τ*_d_ + *τ*_a_. In the model we ignore the synaptic processing time, but the results are not affected by this assumption. Subtracting the two equations gives the evolution equation for the relative phase of the two neurons:


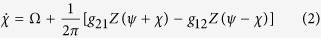


where we assumed that the propagation delay *ψ*_12_ = *ψ*_21_ = *ψ* is identical in both directions. *χ* = *φ*_2_ − *φ*_1_ denotes the phase lag between oscillators, and Ω = *ω*_2_ − *ω*_1_ is the frequency mismatch of the oscillators.

Assuming that pre- and postsynaptic neurons fire at *t*_*j*_ and *t*_*i*_, respectively, the effect of the spikes is received by the synapse at the times *t*_*j*_ + *τ*_a_ and *t*_*i*_ + *τ*_d_ (see Fig. 1A). Therefore, the difference of spikes timing of two neurons at the synaptic site is Δ*t* + *ξ*, where Δ*t* = *t*_*i*_ − *t*_*j*_ is the difference of spike timings of post- and presynaptic neurons at cell body, and *ξ* = *τ*_d_ − *τ*_a_ is the difference of axonal and backpropagation delays which the latter is assumed to be equal to the dendritic forward propagation time *τ*_d_. Dynamical equations of the evolution of synaptic strengths through pair based additive STDP are then:


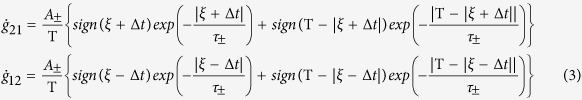


where *A*_+_ (*A*_−_) and *τ*_+_ (*τ*_−_) are the rate and the effective time window of synaptic potentiation (depression), respectively and sign() is the so-called sign function. Note the sum of the dendritic and axonal delays enters the equations describing the neural dynamics (*ψ* in equation (2)), and their difference determine the synaptic dynamics (*ξ* in equation (3)). We take a balanced profile *A*_+_ = *A*_−_ and *τ*_+_ = *τ*_−_7 to better clarify the effect of delay times.

The core idea of the present study is demonstrated in Fig. 1. For a pair of reciprocally coupled neurons, depending on the dendritic and axonal delays and the time lag between spike timing of the two neurons, different patterns of relative timing might occur at the two synapses which can be different from the ordering of spikes at the cell bodies of the two neurons (Fig. 1B and D). During two successive spikes of neuron 1, for example, the synaptic potentiation and depression terms compete in equation (3) to determine the net synaptic change over a period. Ignoring propagation delay times, the distribution of pre- and postsynaptic spikes for one synapse is inverse of that of the other synapse (Fig. 1C). Therefore, for a balanced profile of STDP the potentiation of one synapse is complemented by the same amount of depression of the other synapse (Fig. 1F). Ultimately this leads to elimination of all two-neuron loops and only unidirectional connections can survive^12^,^20^,^21^, regardless of the distribution of the relative spiking times. With more biologically valid STDP profiles, where *A*_+_ > *A*_−_ and *τ*_+_ < *τ*_−_ (with *A*_+_*τ*_+_ < *A*_−_*τ*_−_)^1^, the final structure depends on the distribution of spike times. Bidirectional connections can be maintained if the neurons are almost inphase (the peaks in two distributions are close to zero) and the distributions are wide enough. This result relies on the larger gain of the potentiation part of the STDP profile for near synchronous causal firing of pre- and post synaptic neurons (*A*_+_ > *A*_−_) and can explain how jitters in the locked state of coupled neurons can lead to potentiation of bidirectional connections^62^. In the other limit, uncorrelated firing of the two neurons with flat distribution of the relative spike times leads to elimination of both connections since the commutative change of both synapses is negative due to the condition *A*_+_*τ*_+_ < *A*_−_*τ*_−_. The formation of bidirectional and uncoupled final structures has also been shown to be feasible with potentiation- and depression dominated STDP, respectively, with identical time constants of potentiation and depression^21^. All mentioned results apply when the dendritic and axonal delays are identical since their difference is the quantity which enters in the equations determining the synaptic changes.

**Figure 2:**
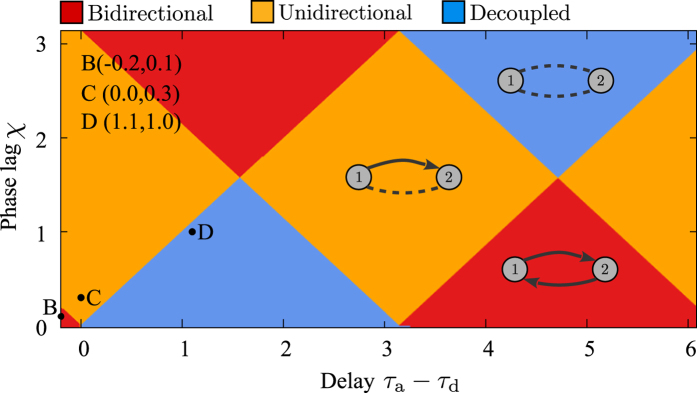
Phase lag and propagation delays determine synaptic modifications. Given the difference of dendritic and axonal propagation delays and assuming that the phase lag is a free parameter, the sign of the synaptic modification and the final structure of the motif can be predicted. Colors show which of the three possible final patterns emerge. The points B–D are the points corresponding to the Fig. 1B–D.

Taking into account the delays and assuming the time lag of spiking is a free parameter, it is easy to check that either of the cases potentiation-depression, potentiation-potentiation, or depression-depression are possible (Fig. 2) without the constraints stated above. With a balanced STDP profile (*A*_+_ = *A*_−_ and *τ*_+_ = *τ*_−_), the sign of net change over one period for each synapse is determined through equation (3) (depicted by 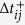 and 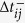, *i, j* = 1, 2, *j* ≠ *i* in Fig. 1B–D). Therefore, depending on the phase lag and propagation delay times it can be determined whether each synapse is potentiated or depressed over one period and if the neurons are (almost) phase-locked, the pattern of spikes is repeated and the synaptic changes build up to determine the final configuration of the motif. Ignoring the delays, or when dendritic and axonal delays are identical *ξ* = 0, reciprocal connections can be jointly potentiated only with wide distribution of relative spike times and fairly larger potentiation gain for small time lags, and simultaneous depression of reciprocal connections is only possible when the neurons are uncorrelated and the average depression is dominated^12^,^21^,^62^. This latter argument might shed additional doubt on the notion that decoupling the neurons is possible by decorrelating their activity by noisy stimulation since, as will be shown below, the depression of both reciprocal synapses is possible in a phase-locked state, even in the noise-free condition (see also^62^).

The next step in our study is to derive the phase lag of the spiking of the neurons through equation (2). With the reasonable assumption that the rate of synaptic change *A*_±_ is small, and the changes in synaptic strength are negligible on the fast time scale of the system 1/*ω*, equation (2) can be solved to obtain stable phase lag of the spiking of the two neurons with constant synaptic strengths. With the gradual changes in synaptic strengths the system is assumed to remain in the fixed point of equation (2) so the relative phase of spiking of the two neurons is determined by the equation (2) which itself determines the gradual change in synaptic strengths according to equation (3). This allows to fully determine the dynamics of the system of equations (2) and (3) knowing the delay times and initial values of the synaptic strengths.

### Two-neuron motif

**Figure 3:**
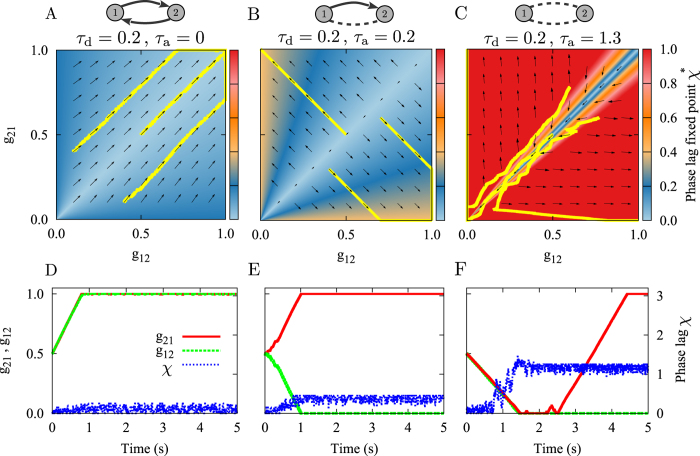
Theoretical prediction of synaptic modifications. (**A–C**) The colors show the phase lag of spiking of the neurons derived from equation (2) and the vector field shows the direction of the change in synaptic strengths from equation (3). The yellow curves denote the simulated synaptic evolution for three different initial values. Based on the delay times the emergent structure can be a bidirectional connection (**A**), unidirectional connection (**B**), or the neurons are decoupled (**C**). (**D–F**) Time course of simulated synaptic strengths (green and red) and phase lag (blue) with the same parameters used in panels (**A–C**), respectively. The dendritic propagation delay is fixed at *τ*_d_ = 0.2. STDP parameters are *A*_+_ = *A*_−_ = 0.005, and *τ*_+_ = *τ*_−_ = 1. The initial values of the synaptic strengths are *g*_21_(0) = *g*_12_(0) = 0.5.

Using the theoretical background presented in the previous section, we investigated how propagation delays affect the configuration of the connections in an initially bidirectionally coupled two-neuron motif. We have solved equation (2) for constant *g*_12_ and *g*_21_ for two canonical forms of infinitesimal phase sensitivity functions for type-I and type-II neurons (see Methods). The results shown in Fig. 3 are drawn for different values of normalized difference of synaptic strengths. For a symmetric configuration with *g*_12_ = *g*_21_ only inphase and antiphase spikings are stable due to the total propagation delay time *τ*_*ij*_. For asymmetric configurations the phase lag is determined by the delay time and difference of the synaptic strengths. The resulted phase lag along with the difference of dendritic and axonal delays determine changes in synaptic strengths through equation (3). We assume that the value of dendritic delay is small (*τ*_d_ = 0.2) and the axonal delay is ranging from 0 to the period of the oscillations (T = 2*π*). The choices in the normalized scale are consistent with experimental measurements of axonal propagation delays in cortico-cortical connections^63^. The results are summed up in Fig. 3A–C: The colors show the stable phase lag derived from equation (2) and the vector field (arrows) shows the changes of synaptic strengths (equation (3)). Given the initial values of the synaptic strengths, the instantaneous (color coded) phase lag determines the synaptic changes, depicted by the vector field (arrows), and the subsequent values of synaptic strength. All three possible final structures can be achieved depending on the delay times and response function of the neurons (in Fig. 3 the results for type-II neurons are presented). The corresponding trajectories of the synaptic strength resulting from the numerical experiments with the three different initial values shown by solid lines in Fig. 3A–C fairly follow the vector field lines predicted by the analytical results.

Time courses of phase lag and synaptic strength, are shown in Fig. 3D–F for several exemplary values of the delay. Our numerical experiments with conductance-based models support that the results are qualitatively valid in more realistic models (see Supplementary Figs S1 and S2). The results show that even for a balanced profile of STDP which is believed to always lead to unidirectional connections, final structures can be bidirectional connections or uncoupled neurons. In either case, the presented theory can predict the final structure if the response curve of the neurons and dendritic and axonal delays are known. Note the results presented in Fig. 3 were obtained with a balanced STDP profile and in the regime of locking with small jitters due to the small amplitude external noise. Hence, the formation of different configurations is purely an effect of considering delays in the model. The only point about decoupling the neurons through STDP is that the decoupled configuration is not stable and a unidirectional coupling eventually emerges in a long-term simulation as is shown in Fig. 3F. This point will be clarified below.

**Figure 4:**
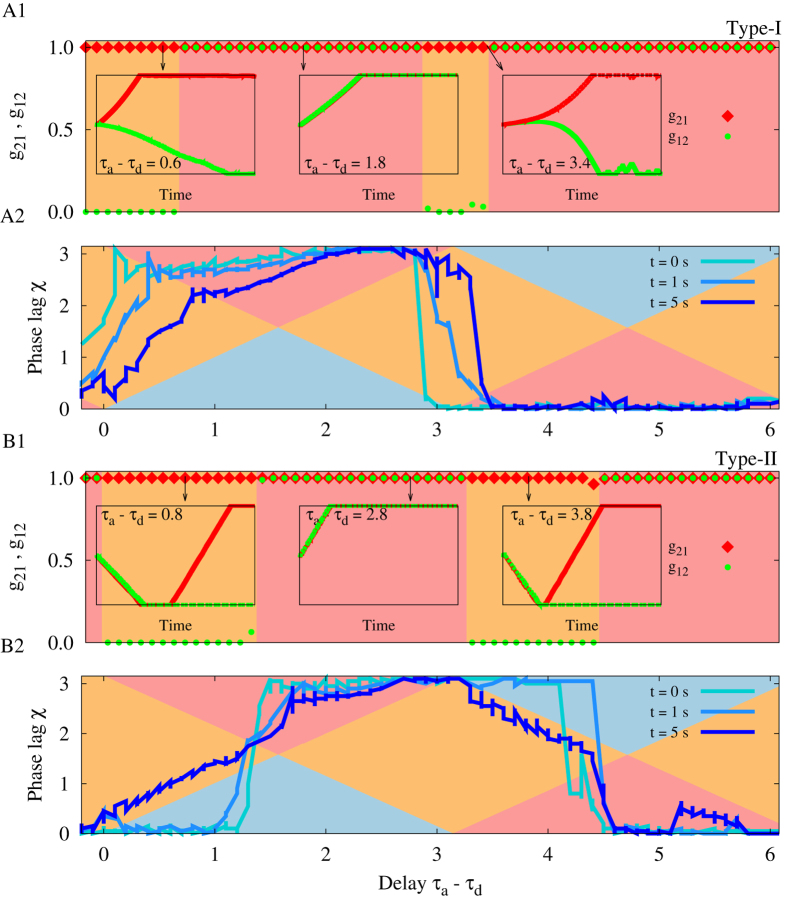
The role of the type of excitability and the PRC of neurons in the evolution of synaptic strengths. (**A1,B1**) Show the simulation results for final synaptic strengths vs. difference of dendritic and axonal propagation delays for typical type-I (A1) and type-II (B1) neurons. The red and green signs show the final values of the synaptic strengths and the background colors illustrate whether a unidirectional (orange) or bidirectional (red) configuration is formed. In the insets three different time courses of the synaptic strengths are shown and in particular it is shown that initial depression of both synapses ultimately leads to a unidirectional configuration. (**A2,B2**) Three snapshots of the time lag of spiking have been shown. Background colors are those in Fig. 2 showing the theoretical prediction of the evolution of the synaptic strengths. If the phase lag (sequential blue curves) lies in the range with orange, red, and blue background color; a unidirectional, bidirectional, and decoupled structure is expected, respectively. If the time lag crosses the intersection of the regions, the direction of the evolution of the synapses changes as is shown in the leftmost inset plot of (B1). In the simulations the dendritic propagation delay is fixed at *τ*_d_ = 0.2. STDP parameters are *A*_+_ = *A*_−_ = 0.005, and *τ*_+_ = *τ*_−_ = 1. The initial values of the synaptic strengths are *g*_21_(0) = *g*_12_(0) = 0.5.

The spiking phase lag in the locked state for two coupled neurons depends on the type of excitability of the neurons and, more generally, on their response function^55^,^56^. Since the phase lag is the determinant of the synaptic evolution, the final configuration of the motif is also affected by the PRC of the neurons. In Fig. 4 we show the evolution of the synaptic strengths and the phase lag for two different PRCs which are typical for canonical forms of type-I and type-II neurons. The plots are superimposed on the Fig. 2 to confirm that the predictions based on the theoretical arguments are valid: depending on the temporary values of the phase lag, the background colors predict the resultant value of the two connection strengths which are mostly consistent with the numeric results for the final synaptic strengths. It is also evident that the final structure of the motif for different values of delay times is also affected by the intrinsic properties of neurons characterized by their PRC.

Notably in our simulations the type-I neurons had not been decoupled for any value of the delay time, and for type-II neurons in a range of delay time the neurons are first decoupled and eventually one of the synapses gets potentiated to result in a unidirectional configuration. For type-I neurons the results are compatible with theoretical expectations: the regions with blue background colors (which lead to depression of both synapses) need small phase lag (close to inphase firing) for small axonal delays and large phase lag (close to antiphase firing) for large delays, while the dynamics of the system of type-I neurons is exactly reverse of this requirement (see Fig. 4A2). For type-II neurons for small values of axonal delays (with *τ*_a_ − *τ*_d_ > 0) neurons initially fire inphase (see Fig. 4B2), and the theory predicts that initially both synapses should be depressed. But depression of the synapses results in large fluctuations of the phase difference due to the small amplitude external noise which can lead to stochastic asymmetric changes in synaptic strengths, which changes (increases) the phase lag such that the system moves from the blue region (depression of both synapses) to the orange region (potentiation of one of the synapses) and ultimately a unidirectional configuration is formed (see Fig. 4B1). Note, in our case the phase lags are determined by the connections within the system and in the case that spikings (and their timing difference) are controlled by external stimulation, the decoupled configuration can be achieved and maintained. This result could be of importance in treatment methods for neurological disorders by external stimulation^64^,^65^,^66^.

### Impact on recurrent networks

It is well-known that in the absence of independent noisy input^24^ STDP leads to an elimination of two-neuron loops in neuronal networks^12^,^20^,^21^ due to the elimination of bidirectional connections through STDP. Based on the results of previous sections, we hypothesize that in the presence of propagation delays in an ensemble of neuronal oscillators this rule no longer holds. To this end, we consider a network of 100 excitatory neurons with all to all connectivity. Initial values of the synaptic strengths are picked from a narrow Gaussian distribution with mean 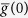 and standard deviation *δg*. Delays (dendritic and axonal) are assumed identical for all synapses. We study how the mean connection strength, distribution of synaptic strengths, and number of two-neuron loops change in the network through STDP. The results of a two-neuron motif are a guide to predict the emergent structure of an entire network^67^. For example, it is expected that parameters which led to potentiation of both connections in the motif (Fig. 3A), potentiate all connections and retain the loops number in the network; while parameters which result in an opposite change eliminate two-neuron loops in the network.

**Figure 5:**
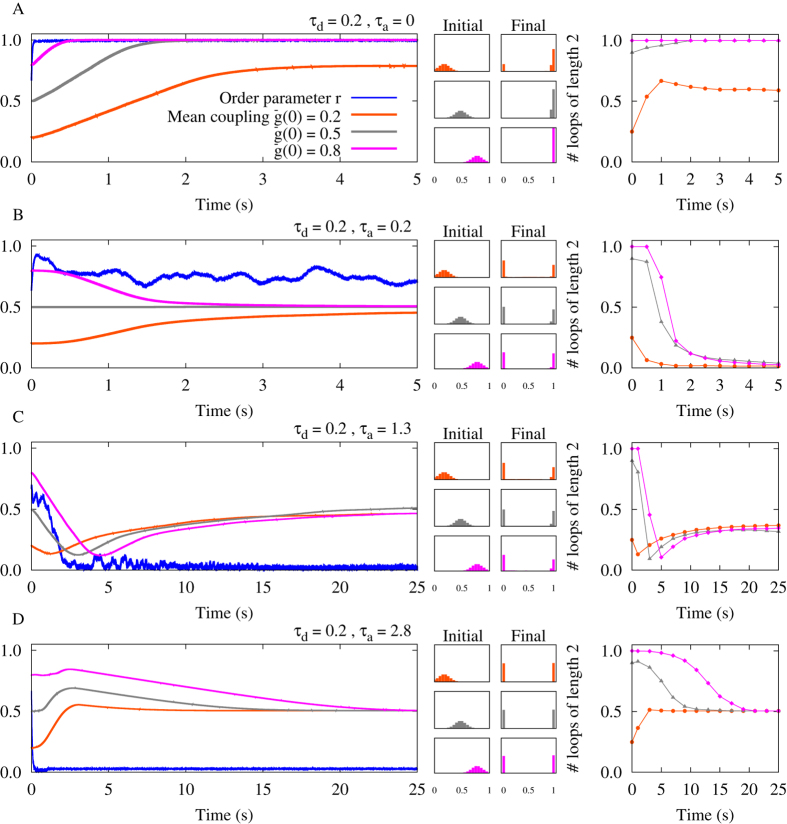
Simulation results for a network of N = 100 type-II phase oscillators. Left panels show the order parameter and three different mean couplings belonging to different distribution of synaptic strengths. Middle panels denote corresponding initial distributions: Gaussian distribution around the mean value 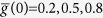 with standard deviation 0.1, and final distribution of synaptic strengths. Right panels indicate the time course of the normalized number of closed loops of length 2 representing the number of bidirectional connections in the network (see Methods). (**A**) Inphase firing. Different distributions of synaptic strength lead to a collective potentiation of the synaptic strengths. The number of loops reaches its maximum value. (**B**) Nearly inphase firing. STDP eliminates strong recurrent loops between neurons and leads to unidirectional connections. (**C**) All connections are decoupled and loops are eliminated. (**D**) Antiphase firing. STDP potentiates recurrent loops while weakening the other connections. In the simulations, the initial values of phases are uniformly distributed between 0 and *π*. The dendritic propagation delay is fixed at *τ*_d_ = 0.2. STDP parameters are *A*_+_ = *A*_−_ = 0.005, and *τ*_+_ = *τ*_−_ = 1.

Results shown in Fig. 5 are produced with the same parameters used in Fig. 3. The results of Fig. 5A-C are in accordance with our aforementioned hypothesis: the loops are all maintained and the mean synaptic strengths increased in Fig. 5A due to the potentiation of all reciprocal synapses. On the other hand, in Fig. 5B bidirectional connections are mainly eliminated, while the mean synaptic strength approaches half of its maximum possible value since from each pair of bidirectional connections one of them is potentiated and the other is eliminated. Fig. 5C shows the situation where all synapses in the network get depressed. As argued in the previous section, this state is highly unstable since after initial depression of the synapses, the system is vulnerable to noise and stochastic changes in the synaptic strengths, usually leading to the state of unidirectional connections like in Fig. 3C.

However, we have to be careful when generalizing the predictions based on the results of two-neuron motifs to the entire network. In the parameter range within which the connections are *repulsive* (i.e. where the anti-phase state is stable for the two-neuron system), the results of the two-neuron motif are not applicable to the entire network as numerical experiments show in Fig. 5D. This is because the connectivity of the interconnected network does not support the retention of the *π* phase difference through all links^68^. Hence, in contrast to the inphase state, the antiphase state does not constitute a building block for the entire network. In fact, antiphase connections cannot be retained when the motifs are put together in a network with dense connections. This can be illustrated by considering a three-neuron loop around which the sum of phase differences should be multiples of 2*π* and this is at odds with the presence of a *π* phase difference in all three links of the loop. Such a geometric constraint on the relative phase relations between neurons in systems of this kind leads to *frustrated dynamics*^68^. In this case, the relative phase relations between neurons and consequently the changes in the network connectivity through STDP cannot be readily predicted by the analysis of two-neuron motif. Our results also show that in this system the final steady state is achieved on a much longer time. Small changes in synaptic constants in the frustrated systems can result in a considerable change of the configuration of the phase lags and consequently the transient time for the frustrated systems is considerably longer than for the synchronized systems.

## Discussion

While the original statement of Hebb’s postulate^69^ controls the modification of the synapses due to the causal relationship between the activity of the neurons, this fact was not sufficiently taken into account in early computational studies of Hebbian plasticity which used correlation of the activity of the neurons leading to symmetric modifications of the reciprocal synapses^70^. Later on experiments showed that the synapses evolve based on the time ordering of the spiking activity in an asymmetric manner^1^ leading to the revival of Hebb’s original postulate which overtly stresses the impact of causality of the activity of neurons on the synaptic changes^70^. STDP not only strengthens the synapse when the presynaptic spike precedes the postsynaptic one, but also penalizes the synapse in the reverse direction. Moreover, the STDP rule is basically a positive feedback procedure. Downstream neurons in the route of causal activities are more likely to be activated after the firing of the upstream neurons and this further strengthens the forward connections in this direction, while the synapses in the reverse direction are weakened. This asymmetric positive feedback modification of synapses results in an instability and renders networks dysfunctional^18^.

Several variations of the synaptic modification rule have been proposed to seek agreement between experimental results and theoretical predictions^9^,^11^,^19^,^21^. Here we studied how considering dendritic and axonal delays in the STDP model can change the well-known effects of conventional STDP on the connecting structure of neuronal networks. Propagation delays affect the synaptic modification through two different ways: The sum of the dendritic and axonal delays is one of the pivotal parameters to determine the time difference of spiking of the coupled neurons, and the difference of these two delay times enter the synaptic modification equations since the effect of pre- and postsynaptic spikes are not instantaneously received at the synaptic site. In particular, we have shown that both unidirectional and bidirectional connections can emerge in different ranges of the delay times.

Our result also show that the joint depression of reciprocal connections between neurons is possible if the initial connections are of almost the same strength. However, by the resultant weak connections the system can no longer maintain the neurons in the phase-locked state, and the fluctuations of the phase difference in this case lead to unidirectional connections. Yet, the possibility of the simultaneous depression of the reciprocal connections in a phase-locked state can shed light on how coordinated stimulation of different brain regions might lead to an unlearning of the pathological synchronized dynamics^65^,^66^.

Previous studies showed that the pairwise analysis can predict the structure of recurrent populations^67^. Our study shows that the generalization of the results of the two-neuron motif to the network is possible when the connections are synchronizing, i.e. when they induce inphase spiking of the neurons. In this case, we have shown that through STDP with balanced profile and in the absence of noise, the neuronal loops can be maintained through joint potentiation of the reciprocal connections. This result challenges previous results on the effect of conventional STDP which was supposed to eliminate loops in recurrent networks^7^,^12^,^20^,^21^,^40^. On the other hand, we have shown that the results of the two-neuron motif cannot predict the evolution of the structure of neural populations when the connections are repulsive, i.e. when the reciprocal connections give rise to antiphase spiking of the neurons. In these systems the presence of multiple competing connections on each neuron, makes it impossible to predict the relative dynamics of the neurons in the network based on the results of two-neuron motifs. Our theory relies on the calculation of the instantaneous phase lag of the spiking of the neurons in a network with slowly varying connections, which fails to predict phase lags in such *frustrated* networks^68^. The rich dynamics of frustrated networks is hard to asses even in a static network and is beyond of the scope of the present study.

Note, our study was performed for coupled neurons and networks in the absence of noise. However, independent noise can induce strong bidirectional synaptic coupling that is absent in the noise-free situations as shown in large systems of oscillatory Hodgkin-Huxley neurons and phase oscillators^62^ and in just two coupled neurons^24^. In these studies it was shown that the mean synaptic weight increases in a stochastic resonance-like manner. In this way, STDP constitutes a natural resistance to noise^62^,^71^. Future studies should be devoted to the interplay of dendritic and axonal propagation delays on the one hand and independent noise on the other hand.

Ultimately, the possibility of a simultaneous depression of reciprocal connections in a phase-locked state may contribute to a further development of brain stimulation techniques that cause an anti-kindling, i.e. an unlearning of abnormally up-regulated synaptic connectivity and, in turn, abnormal synchrony^64^. In fact, coordinated reset (CR) stimulation^72^, a desynchronizing multi-site stimulation technique was successfully tested in pre-clinical^73^ and clinical^74^,^75^ proof of concept studies. However, based on the approach presented here, further improvements might be achievable.

## Methods

### Spike-timing-dependent plasticity (STDP)

The neuronal oscillators are subjected to STDP where the synaptic strengths *g*_*ij*_ = *g*_*ij*_(*t*) are updated by an additive update rule in each step, *g*_*ij*_ → *g*_*ij*_ + Δ*g*_*ij*_(Δ*t*_*ij*_) according to the following STDP function:


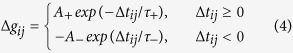


where Δ*t*_*ij*_ = *t*_*i*_ − *t*_*j*_ is the time lag between presynaptic neuron *j* and postsynaptic neuron *i. A*_+_ (*A*_−_) and *τ*_+_(*τ*_−_) are the rate and the effective time window of synaptic potentiation (depression), respectively. The synaptic strengths are confined to the interval [*g*_min_, *g*_max_] = [0, 1] and parameters are set to *A*_+_ = *A*_−_ = 0.005, and *τ*_+_ = *τ*_−_ = 1 in case of the phase oscillator model. In case of conductance-based models (see Supplementary), the synaptic strengths are confined to the range [*g*_min_, *g*_max_] = [0, 0.2] mS/cm^2^, and we consider parameters *A*_+_ = *A*_−_ = 0.005 mS/cm^2^, and *τ*_+_ = *τ*_−_ = 20 ms. It should be noted that hard boundaries are imposed on the allowed range of synaptic strengths. The synaptic strengths are set to *g*_min_ (*g*_max_) as soon as they cross the lower (upper) limit of their allowed range.

### Phase model for weakly pulse-coupled oscillators

The general form of many weakly pulse-coupled oscillators in terms of the phases of the oscillators can be written as follows^76^:


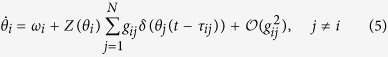


where *θ*_*i*_ is the phase and *ω*_*i*_ is the natural frequency of the oscillations. *Z*(*θ*_*i*_) is the PRC of the corresponding oscillator. *δ*(*θ*_*j*_) is the so-called Dirac’s delta function indicating the pulsatile interactions between coupled oscillators. *τ*_*ij*_ is the total propagation delay between two oscillators. One can represent the phase of the oscillations, *θ*_*i*_ in terms of *ϕ*_*i*_, the slowly changing phase deviating from the natural fast oscillation term *ω*_*i*_*t* as *θ*_*i*_(*t*) = *ω*_*i*_*t* + *ϕ*_*i*_. Note, *Z*(*ω*_*i*_*t* + *ϕ*_*i*_) is a *T*_*i*_-periodic function, and the scaling of the pulsatile term of oscillations by the small parameter *g*_*ij*_ indicates that changes in the relative phases *ϕ*_*j*_ occur on a much slower timescale than *T*_*i*_. Therefore, one can integrate the pulsatile term over the full period *T*_*i*_ holding the values of *ϕ*_*j*_ constant to obtain the average rate of change of *ϕ*_*j*_ over a cycle. The averaging theory provides a near-identity change of variables as 
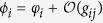
. These assumptions finally transforms equation (5) to the reduced phase model of equation (1). The 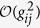 term can be ignored due to the small changes of the parameter *g*_*ij*_. For more details see ref. 77.

### Dynamical analysis of the joint phase model

Assuming that the frequency mismatch between the two oscillators is negligible 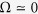, the fixed point χ^*^ of the phase lag of equation (2) for type-I PRC with 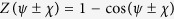 is:


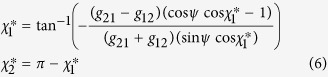


where 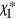 is for inphase firing and 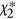 belongs to the antiphase state. Knowing the synaptic strengths, only one of these fixed points are stable in a given delay time *ψ*. Equation (6) shows that the fixed points of type-I oscillations are self-consistent. In this case the 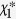 is simply where the two 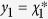 and 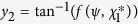 curves intersect. The other approach is to solve the equation 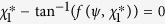 numerically, using any root-finding scheme. On the other hand, the fixed points of type-II oscillations are rather straightforward. The fixed point of phase lag for type-II PRC with 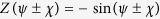 can be derived similarly:


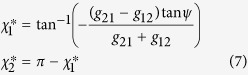


### Network model

A fully-connected network of *N* = 100 excitatory type-II phase oscillators was simulated. The phase oscillators obey equation (1), and the synaptic strengths are modified by the STDP profile according to equation (4). Initial values of synaptic strengths are Gaussian distributed around the mean value 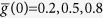 with standard deviation 0.1. The phases of the oscillators are initially uniformly between 0 and *π*. The dendritic propagation delay is fixed at *τ*_d_ = 0.2. STDP parameters are *A*_+_ = *A*_−_ = 0.005, and *τ*_+_ = *τ*_−_ = 1. We also define an order parameter *r*(*t*), for the network of *N* = 100 phase oscillators ranging between 0 and 1, that measures the degree to which the system is synchronized:


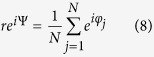


where Ψ(*t*) is the average phase^78^.

### Counting loops

In this study a bidirectional connection corresponds to a closed loop of length *n* = 2 in a network of neuronal phase oscillators. In order to measure the number of such closed loops, we construct a directed graph^20^. Transformation of the strength matrix ***G*** into a directed graph is performed by considering a threshold *h* = 0.2^20^,^21^. Assuming that there are no self-loops (i.e. *g*_*ii*_ = 0), the corresponding value in the adjacency matrix ***M*** of the resultant directed graph is assigned to 1 whenever the synaptic strength is greater than *h*, and is assigned to zero otherwise. Therefore the number of closed loops of length *n* = 2 in the adjacency matrix ***M*** is:


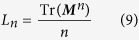


where Tr denotes the matrix trace. In Fig. 5 (right panels), in order to perform a better comparison, this quantity is normalized to the total number of possible loops of the same length i.e. *N*(*N* − 1)/2, ignoring self-loops, where *N* denotes the number of the phase oscillators or nodes in the network. Therefore the result is a normalized number between 0 and 1.

## Additional Information

**How to cite this article**: Madadi Asl, M. *et al*. Dendritic and Axonal Propagation Delays Determine Emergent Structures of Neuronal Networks with Plastic Synapses. *Sci. Rep.* **7**, 39682; doi: 10.1038/srep39682 (2017).

**Publisher's note:** Springer Nature remains neutral with regard to jurisdictional claims in published maps and institutional affiliations.

## Supplementary Material

Supplementary Information
