# Supplementary material for: Dendritic and Axonal Propagation Delays Determine Emergent Structures of Neuronal Networks with Plastic Synapses
Source: Sci Rep. 2017 Jan 3;7:39682. doi: 10.1038/srep39682 (PMC5206725; doi:10.1038/srep39682)
Supplement: Supplementary Information [file srep39682-s1.pdf]

Supplementary Information for:
Dendritic and Axonal Propagation Delays Determine Emergent Structures of Neuronal Networks with Plastic Synapses

Mojtaba Madadi Asl, Alireza Valizadeh, and Peter A. Tass

Neuronal and synaptic models

We consider two well-known conductance-based neuronal models: Wang-Buzsáki (WB) [79] and Hodgkin-Huxley (HH) [80] neurons that mimic the behavior of type-I and type-II oscillators, respectively. The time evolution of the voltage of both WB and HH models governs the following equation:

$$C\frac{dV_i}{dt} = I_{\mathrm{app}} - I_{ij}(t) - \bar{g}_{\mathrm{Na}}m^3h(V_i - V_{\mathrm{Na}}) - \bar{g}_{\mathrm{K}}n^4(V_i - V_{\mathrm{K}}) - \bar{g}_{\mathrm{L}}(V_i - V_{\mathrm{L}}) \tag{S1}$$

where j and i denote the indices of pre- and postsynaptic neurons, respectively. C is the membrane capacitance in $\mu\mathrm{F/cm}^2$, V_i is the membrane voltage in mV. $\bar{g}_{\mathrm{Na}}$, $\bar{g}_{\mathrm{K}}$, and $\bar{g}_{\mathrm{L}}$ are maximal conductances of sodium, potassium and leak currents in $\mathrm{mS/cm}^2$. V_{Na}, V_{K} and V_{L} are corresponding Nernst equilibrium potentials in mV. The numerical values of these parameters that we used in our simulations are listed in Table **S1**. The parameters n, m and h are gating variables in which satisfy equations (S2) and (S3) in case of WB and HH conductance-based models, respectively [79,80]:

$$\begin{aligned}
&dn(t)/dt = \phi\,(\alpha_n(V)(1-n(t)) - \beta_n(V)n(t))\\
&dh(t)/dt = \phi\,(\alpha_h(V)(1-h(t)) - \beta_h(V)h(t))\\
&m(t) = m_\infty(V) = \alpha_m(V)/(\alpha_m(V)+\beta_m(V))
\end{aligned} \tag{S2}$$

$$\begin{aligned}
&dn(t)/dt = \alpha_n(V)(1-n(t)) - \beta_n(V)n(t)\\
&dh(t)/dt = \alpha_h(V)(1-h(t)) - \beta_h(V)h(t)\\
&dm(t)/dt = \alpha_m(V)(1-m(t)) - \beta_m(V)m(t)
\end{aligned} \tag{S3}$$

where the functions $\alpha(V)$ and $\beta(V)$ describe the transition rates between open and closed states of the channels in which satisfy the equations (S4) and (S5) in case of WB and HH models, respectively [79,80]:

$$\begin{aligned}
&\alpha_n(V) = -0.01(V+34)/(exp(-0.1(V+34))-1)\\
&\beta_n(V) = 0.125\,exp(-(V+44)/80)\\
&\alpha_h(V) = 0.07\,exp(-(V+58)/20)\\
&\beta_h(V) = 1/(exp(-0.1(V+28))+1)\\
&\alpha_m(V) = -0.1(V+35)/(exp(-0.1(V+35))-1)\\
&\beta_m(V) = 4\,exp(-(V+60)/18)
\end{aligned} \tag{S4}$$

$$\begin{aligned}
&\alpha_n(V) = (0.1-0.01\,V)/(exp(1-0.1\,V)-1)\\
&\beta_n(V) = 0.125\,exp(-V/80)\\
&\alpha_h(V) = 0.07\,exp(-V/20)\\
&\beta_h(V) = 1/(exp(3-0.1\,V)+1)\\
&\alpha_m(V) = (2.5-0.1\,V)/(exp(2.5-0.1\,V)-1)\\
&\beta_m(V) = 4\,exp(-V/18)
\end{aligned} \tag{S5}$$

I_{app} in equation (S1) is the injected current to the neuron i in $\mu\mathrm{A/cm}^2$. We assume that neurons are connected to each other by excitatory chemical synapses. $I_{ij}(t)$ is the synaptic current from presynaptic to postsynaptic neuron, given by:

Table S1. Neuronal parameters of conductance-based WB and HH models.

**Parameter**	**Symbol**	**WB**	**HH**	**Unit**
Membrane capacitance	C	1	1	μF/cm^2
Spiking threshold	V_{th}	-40	-40	mV
Resting membrane potential	V_{r}	-65	-65	mV
Sodium equilibrium potential	V_{Na}	55	50	mV
Potassium equilibrium potential	V_{K}	-90	-77	mV
Leak equilibrium potential	V_{L}	-65	-54.4	mV
Sodium maximal conductance	$\bar{g}_{\mathrm{Na}}$	35	120	mS/cm^2
Potassium maximal conductance	$\bar{g}_{\mathrm{K}}$	9	36	mS/cm^2
Leak maximal conductance	$\bar{g}_{\mathrm{L}}$	0.1	0.3	mS/cm^2
Applied current	I_{app}	1	10	μA/cm^2

$$I_{ij}(t) = \bar{g}_{ij} s_{ij}(t - \tau_{ij})(V_i - E_{\mathrm{syn}}) \tag{S6}$$

where $\bar{g}_{ij}$ is the maximal synaptic strength, $\tau_{ij} = \tau_{\mathrm{d}} + \tau_{\mathrm{a}}$ is the total propagation delay between pre- and postsynaptic neurons, V_i is the voltage of the postsynaptic neuron i, and E_{syn} is the synaptic reversal potential that characterizes excitatory or inhibitory nature of the synapse. The function $s(t)$ denotes the fraction of open channels and obeys a first-order kinetics [79]:

$$\frac{ds_{ij}}{dt} = \alpha f(V_j - V_{\mathrm{th}})(1 - s_{ij}) - \beta s_{ij} \tag{S7}$$

where α and β are channel opening and closing rates in ms^{-1}, respectively. The function $f(V_j) = 0.5[1 + \tanh(\eta V_j)]$ guarantees activation of the synapse whenever the presynaptic voltage crosses V_{th} and the parameter η is a constant.

Simulation results for conductance-based models

In order to evaluate our developed framework in more realistic models, we considered two WB and HH conductance-based neurons that are reciprocally connected to each other by symmetric excitatory chemical synapses. WB and HH neurons mimic the behavior of type-I and type-II oscillators, respectively. In particular, the PRC of WB and HH neurons is qualitatively analogous to the response function of type-I and type-II oscillators. This suggests that the distribution of time lags and in a result the synaptic strengths dynamics of two WB and HH coupled neurons are generally similar to that of type-I and type-II oscillators, respectively. Therefore, one can expect that the results presented in Fig. 4 for type-I and type-II oscillators are qualitatively valid in case of WB and HH neurons.

Figs. **S1** and **S2** show the simulation results for WB and HH conductance-based models, respectively. The simulation results for final synaptic strengths presented in Figs. **S1**D and **S2**D are generally consistent with results illustrated in Fig. 4A1 and B1 for type-I and type-II oscillators, respectively. Figs. **S1**A-C and **S2**A-C show the time course of simulated synaptic strengths (green and red) and time lag (blue) for different values of axonal propagation delay when the dendritic propagation delay is constant. All three possible final configurations of synaptic strengths can be achieved depending on the delay times and response function of the neurons. This strongly illustrates that our results are qualitatively valid in more realistic models.

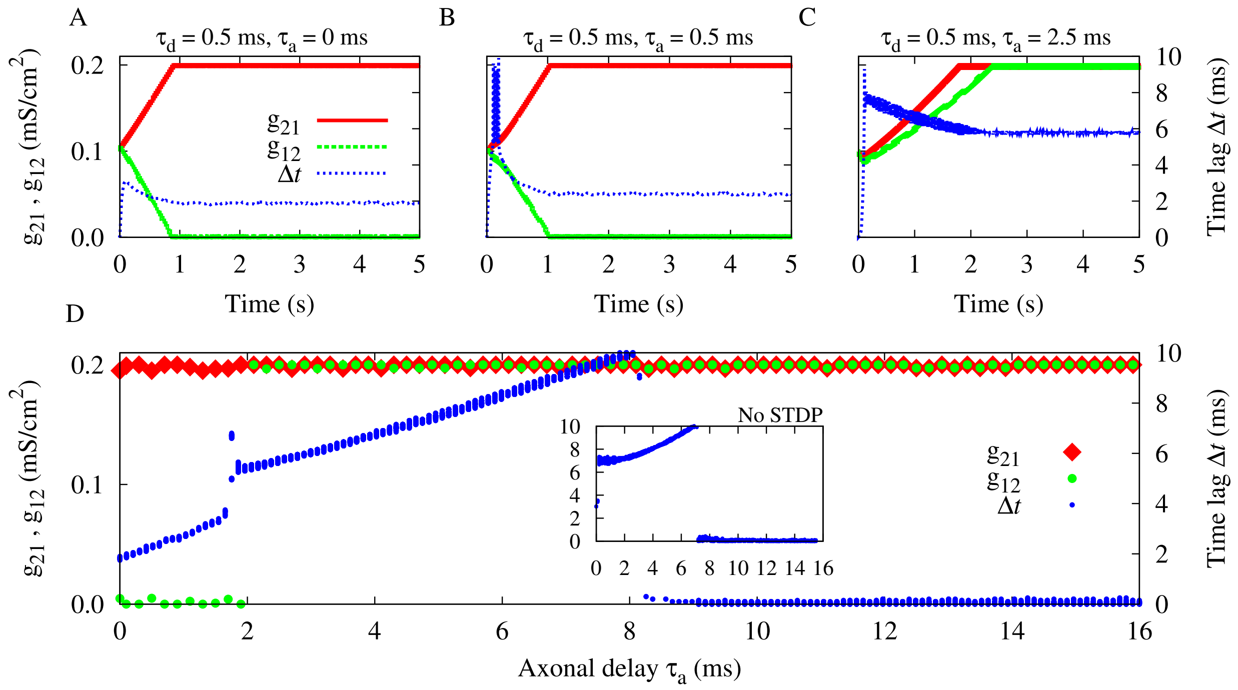

Figure S1. **Simulation results for WB neurons.** (**A-C**). Time course of simulated synaptic strengths (green and red) and time lag (blue). (**D**). Simulation results for final synaptic strengths vs. axonal propagation delay. The inset shows the time lag in the absence of STDP. The dendritic propagation delay is fixed at $\tau_{\rm d} = 0.5\,{\rm ms}$. STDP parameters are $A_+ = A_- = 0.005\,{\rm mS/cm^2}$, and $\tau_+ = \tau_- = 20\,{\rm ms}$. Total time of simulation at a given delay is 10 s. The initial values of synaptic strengths are $g_{21}(0) = g_{12}(0) = 0.1\,{\rm mS/cm^2}$.

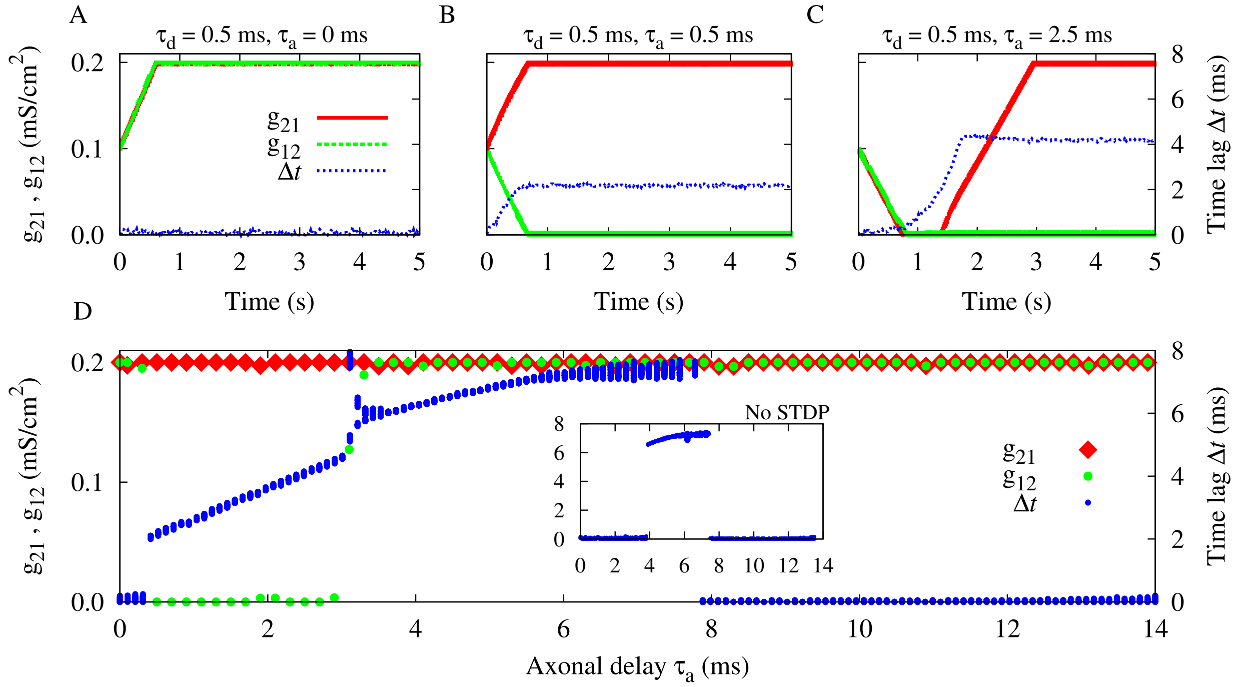

Figure S2. **Simulation results for HH neurons.** (**A-C**). Time course of simulated synaptic strengths (green and red) and time lag (blue). (**D**). Simulation results for final synaptic strengths vs. axonal propagation delay. The inset shows the time lag in the absence of STDP. The dendritic propagation delay is fixed at $\tau_{\rm d} = 0.5\,{\rm ms}$. STDP parameters are $A_+ = A_- = 0.005\,{\rm mS/cm^2}$, and $\tau_+ = \tau_- = 20\,{\rm ms}$. Total time of simulation at a given delay is 10 s. The initial values of synaptic strengths are $g_{21}(0) = g_{12}(0) = 0.1\,{\rm mS/cm^2}$.
